# Immunization With Outer Membrane Vesicles Derived From Major Outer Membrane Protein-Deficient *Salmonella* Typhimurium Mutants for Cross Protection Against *Salmonella* Enteritidis and Avian Pathogenic *Escherichia coli* O78 Infection in Chickens

**DOI:** 10.3389/fmicb.2020.588952

**Published:** 2020-11-03

**Authors:** Yuxuan Chen, Kaiwen Jie, Biaoxian Li, Haiyan Yu, Huan Ruan, Jing Wu, Xiaotian Huang, Qiong Liu

**Affiliations:** ^1^Department of Medical Microbiology, School of Medicine, Nanchang University, Nanchang, China; ^2^The First Clinical Medical College, Nanchang University, Nanchang, China; ^3^Key Laboratory of Tumor Pathogenesis and Molecular Pathology, School of Medicine, Nanchang University, Nanchang, China

**Keywords:** outer membrane vesicle, broad-spectrum vaccine, *Salmonella* Enteritidis, *Escherichia coli* APEC O78, outer membrane protein

## Abstract

Colibacillosis is an economically important infectious disease in poultry, caused by avian pathogenic *Escherichia coli* (APEC). *Salmonella enterica* serovar Enteritidis (*S.* Enteritidis) is a major cause of food-borne diseases in human circulated through poultry-derived products, including meat and chicken eggs. Vaccine control is the mainstream approach for combating these infections, but it is difficult to create a vaccine for the broad-spectrum protection of poultry due to multiple serotypes of these pathogens. Our previous studies have shown that outer membrane vesicles (OMVs) derived from *S. enterica* serovar Typhimurium mutants with a remodeled outer membrane could induce cross-protection against heteroserotypic *Salmonella* infection. Therefore, in this study, we further evaluated the potential of broad-spectrum vaccines based on major outer membrane protein (OMP)-deficient OMVs, including Δ*ompA*, Δ*ompC*, and Δ*ompD*, and determined the protection effectiveness of these candidate vaccines in murine and chicken infection models. The results showed that Δ*ompA* led to an increase in the production of OMVs. Notably, Δ*ompA*Δ*ompC*Δ*ompD* OMVs showed significantly better cross-protection against *S. enterica* serovar Choleraesuis, *S.* Enteritidis, APEC O78, and *Shigella flexneri* 2a than did other *omp*-deficient OMVs, with the exception of Δ*ompA* OMVs. Subsequently, we verified the results in the chicken model, in which Δ*ompA*Δ*ompC*Δ*ompD* OMVs elicited significant cross-protection against *S.* Enteritidis and APEC O78 infections. These findings further confirmed the feasibility of improving the immunogenicity of OMVs by remodeling the outer membrane and provide a new perspective for the development of broad-spectrum vaccines based on OMVs.

## Introduction

*Salmonella* and avian pathogenic *Escherichia coli* (APEC) are both important pathogens that can cause extended and acute bacterial disease, such as avian colibacillosis and fowl typhoid, and even death in poultry, especially in chickens, worldwide (Barrow, 2007; Whiley and Ross, 2015). In addition, as foodborne pathogens, *Salmonella* spp. are also linked to human infections, and direct contact with animals or contaminated poultry meat and eggs can also promote infection with *Salmonella enterica* serovar Enteritidis (*S.* Enteritidis; Bäumler et al., 2000; Barrow, 2007; Singer, 2015). Therefore, *Salmonella* infections are a concern for the global poultry industry and a threat to the health and safety of human populations worldwide. The diseases induced by *Salmonella* and APEC are difficult to control because of numerous serotypes of the pathogens (Gast, 2007; Ebrahimi-Nik et al., 2018). The current strategy mainly relies on the administration of antibiotics against *Salmonella* and APEC (Feasey, 2012). However, the abuse of antibiotics in the poultry industry has led to their low effectiveness and a serious problem of pollution with antibiotic residues (Cavicchio et al., 2015; Subedi et al., 2018; Weiss et al., 2018). In production practice, immunization with multiple or multivalent vaccines is often necessary to obtain a protective effect against these bacteria. However, there still may be an immune failure, which leads to substantial losses in aquaculture and other industries. Therefore, continuous screening for effective vaccine candidates should be performed as a safer and more effective way to improve the effectiveness of cross-protective immunity.

Outer membrane vesicles (OMVs), which are naturally produced by Gram-negative bacteria, have a spherical structure with a bilayer membrane, which contains mainly biologically active components (Kuehn and Kesty, 2005; Ellis and Kuehn, 2010). Bacterial-derived OMVs are heterogeneous complexes, which contain lipopolysaccharide (LPS), lipoproteins, and outer membrane proteins (OMPs), considered to be the prototype of pathogen-associated molecular patterns (Kuehn and Kesty, 2005). In our previous study, we evaluated the immunogenicity of OMVs derived from *S. enterica* serovar Typhimurium (*S.* Typhimurium) with truncated LPS and found that intranasal or intraperitoneal immunization with OMVs from the *waaC* and *rfbP* mutants obviously elicited significantly higher cross-reactive IgG levels and provided increased cross-protection against *S. enterica* serovar Choleraesuis (*S.* Choleraesuis) and *S.* Enteritidis compared with OMVs from other truncated LPS mutations (Liu et al., 2016b). Further, flagellin, as the common contaminant in OMV pellets, affected the purification of OMVs, and resulted in non-essential immune responses, thus weakening the protective immune responses produced by OMVs. Therefore, we investigated the characteristics and protective immune responses of OMVs derived from flagellin-deficient strains. The cryo-EM showed that the Δ*fliC*Δ*fliB* mutations could effectively reduce the contamination of flagellin in the OMVs during density gradient centrifugation, and animal experiments revealed that non-flagellin OMVs provided efficient protection against heterologous *S.* Choleraesuis and *S.* Enteritidis (Liu et al., 2016a). Therefore, exploration of OMV-based vaccines may be an effective approach to vaccine development against homologous and heterologous serotypes of enteropathogenic bacteria.

Outer membrane proteins, as the exposed components on the OMV surface, are potential drug and vaccine targets because of their high immunogenicity and safety (Muralinath et al., 2011; Roier et al., 2012). Moreover, OMPs are anchored to the outer membrane and are essential for maintaining the integrity and selective permeability of bacterial outer membranes (Lin et al., 2002). The knockout of several OMPs could affect the synthesis or assembly of OMPs, resulting in different levels of OMP expression (Charlson et al., 2006). The main OMPs in *Salmonella* are OmpA, OmpC, and OmpD (Grubmeyer et al., 2012). Thus, removing these abundant proteins from the membrane not only leaves more space for recombinant proteins anchored into OMVs as a delivery vaccine but also potentially influences the membrane vesiculation and synthesis of conservative immunogenic OMPs (Meuskens et al., 2017). Therefore, a hypothesis has emerged that remodeling of the outer membrane through deletion of major OMPs may affect the cross-protection with OMVs by affecting the expression of conserved OMPs; the immunogenicity and cross-protection efficacy of OMVs derived from major OMP mutations are subsequently evaluated.

In this study, we constructed various OMP-deficient mutants and assessed the impact of OMP deficiency on the cross-protective efficacy of OMVs in animal experiments. The findings from this study may provide a basis for novel and effective OMV-based broad-spectrum vaccine candidates against reduced infection by *Salmonella* and APEC in chickens for future application in agricultural production.

## Materials and Methods

### Construction of Bacterial Mutant Strains and Cell Culture

Strains of *Salmonella*, *Escherichia coli* APEC O78, and *Shigella flexneri* 2a were routinely grown in Luria-Bertani (LB; Théry et al., 2018) broth or on agar at 37°C. To indicate the reproducibility of isolated OMVs derived from *omp* mutants in different cultures, an acidic low-magnesium minimal medium (acidic MgM), which partially reproduced the intracellular environment, was also used to culture *Salmonella* for purification of OMVs (Yoon et al., 2009; Bai et al., 2014). The bacterial strains and plasmids used in this study are listed in Table 1. To construct the mutant strains, LB agar with 5% sucrose was employed for *sacB* gene-based counterselection (Kong et al., 2011). The growth of Δ*asd* strains required 50 mg/ml diaminopimelic acid (DAP; Nakayama et al., 1988). All primers used in the study are listed in Table 2. *E. coli* was transformed by electroporation, and LB agar plates containing appropriate antibiotics were used to select transformants. We used the suicide plasmid method to construct mutant strains as previously described, and the bacterial strains used were derived from previous experiments (Liu et al., 2016a,b). The resulting PCR product had a terminal A base when using the LA Taq enzyme (TaKaRa, Otsu, Japan), and the amplicon was then inserted into the pYA4278 vector with a terminal T-overhang, generated by *Ahd*I digestion. The same strategy was used to construct pQK014 for *ompA* gene deletion, pQK015 for *ompC* gene deletion, and pQK016 for *ompD* gene deletion. All mutations were introduced into *Salmonella* Typhimurium K084, resulting in a knocked out flagellin and truncated LPS to improve the cross-proteins of OMVs, generating the K021 mutant (Liu et al., 2016a,b).

**Table 1:** Bacterial strains and plasmids used in this study.

Strain or plasmid	Description	Source
**Strains**		
*Salmonella* Typhimurium		
χ3761	*Salmonella* Typhimurium UK-1, LD_50_ = 5.2 × 10^5^ CFU	Liu et al., 2016a
S246	*Salmonella* Enteritidis, LD_50_ = 1.3 × 10^5^ CFU	Liu et al., 2016a
S340	*Salmonella* Choleraesuis, LD_50_ = 1.5 × 10^5^ CFU	Liu et al., 2016a
K083	*Salmonella* Typhimurium χ3761 ∆*fliC* ∆*fljB*	Liu et al., 2016a
K084	*Salmonella* Typhimurium χ3761 ∆*fliC* ∆*fljB* ∆*rfbP*	Liu et al., 2016b
K015	*Salmonella* Typhimurium χ3761 ∆*fliC* ∆*fljB* ∆*rfbP* ∆*ompA*	This study
K016	*Salmonella* Typhimurium χ3761 ∆*fliC* ∆*fljB* ∆*rfbP* ∆*ompC*	This study
K017	*Salmonella* Typhimurium χ3761 ∆*fliC* ∆*fljB* ∆*rfbP* ∆*ompD*	This study
K018	*Salmonella* Typhimurium χ3761 ∆*fliC* ∆*fljB* ∆*rfbP* ∆*ompA* ∆*ompC*	This study
K019	*Salmonella* Typhimurium χ3761 ∆*fliC* ∆*fljB* ∆*rfbP* ∆*ompA* ∆*ompD*	This study
K020	*Salmonella* Typhimurium χ3761 ∆*fliC* ∆*fljB* ∆*rfbP* ∆*ompC* ∆*ompD*	This study
K021	*Salmonella* Typhimurium χ3761 ∆*fliC* ∆*fljB* ∆*rfbP* ∆*ompA* ∆*ompC* ∆*ompD*	This study
*Escherichia coli*		
χ7232	endA1 hsdR17 (rK-,mK+) supE44 thi-1 recA1 gyrArelA1Δ (lacZYA-argF) U169λpirdeoR [φ80dlac Δ (lacZ) M15]	Kong et al., 2011
χ7213	thi-1 thr-1 leuB6 glnV44 tonA21 lacY1 recA1 RP4-2-Tc::μλpir ΔasdA4 Δzhf-2::Tn 10	Kong et al., 2011
O78	Heterogenous infection, LD_50_ = 1.8 × 10^6^ CFU	Lab collection
*Shigella*		
*Shigella flexneri* 2a	Heterogenous infection, LD_50_ = 1.2 × 10^5^ CFU	Lab collection
**Plasmid**		
pYA4278	Suicide vector, *sacB mobRP4 oriR6K* Cm^r^	Kong et al., 2011
pQK014	For deletion of *ompA*	This study
pQK015	For deletion of *ompC*	This study
pQK016	For deletion of *ompD*	This study

**Table 2:** Primers used in this study.

Primers	Sequences (5'-3')	Function
ompA-1F	CATCCTCTCACACAACGAGAC	For deletion of *ompA* gene in *Salmonella*
ompA-1R	CTGCAGGAATGCGGCCGCCGGGGGATCTGCTCAATATT	
ompA-2F	CGGCCGCATTCCTGCAGGTAAGTTATCGTCTGGTAGAAA AAC	
ompA-2R	CATATGAATCCGGAACTGGTC	
ompC-1F	GCCAATACGCAGCGCCGAGGTCACG	For deletion of *ompC* gene in *Salmonella*
ompC-1R	ACCTGCAGGATGCGGCCGCGGTCAGCAAAAGATG	
ompC-2F	CCGCGGCCGCATCCTGCAGGTGTTATTAACCCTCTG	
ompC-2R	ATAGGGGTAAACAGACATTCAGAAG	
ompD-1F	AAAGTTAATGATGATAGCGG	For deletion of *ompD* gene in *Salmonella*
ompD-1R	CTGCAGGAATGCGGCCGCGTTATTAACCCTCTGTTATA	
ompD-2F	CGGCCGCATTCCTGCAGGTAATCTCGATGGATATCGAAC	
ompD-2R	CGTTAAAGCGCATCAGCGCG	

Murine macrophage RAW 264.7 cells were cultured at 37°C with 5% CO_2_ atmosphere in Dulbecco’s modified Eagle’s medium (Gibco BRL, United States) supplemented with 10% fetal bovine serum (HyClone, United States). All experimental protocols were approved by Nanchang University.

### Purification and Protein Profiles of OMVs Derived From Mutant Strains of *Salmonella*

Native OMVs (without any detergent processing) were collected and isolated from culture supernatants of *S.* Typhimurium UK-1 and its mutants, as described previously (Muralinath et al., 2011). Briefly, 2 L bacterial culture supernatants in the logarithmic growth phase (OD_600_ = 1) were collected by filtration through a 0.45-μm Steritop bottle-top filter unit (Millipore, Bedford, MA, United States). Then, the vesicles present in the collected supernatants were pelleted by centrifugation (40,000 × *g*, 4°C, 2 h) and resuspended in Dulbecco’s phosphate-buffered saline (DPBS; Mediatech, Manassas, VA, United States). Further, pelleted OMVs were purified using density gradient centrifugation (200,000 × *g*, 4°C, overnight) with a discontinuous OptiPrep density gradient medium [20, 25, 30, 35, 40, and 45% OptiPrep in 10 mM HEPES (pH 6.8) with 0.85% NaCl, from top to bottom; Sigma-Aldrich, St. Louis, MO, United States].

The concentration of OMVs was determined based on total protein concentration using a bicinchoninic acid assay (Thermo Pierce, Rockford, IL, United States). Each OMV sample (10 μg, based on the protein content) was analyzed by 12% sodium dodecyl sulfate polyacrylamide gel electrophoresis (SDS-PAGE), and the gels were stained using the GelCode™ blue stain reagent (Thermo Pierce).

### Nanoparticle Tracking Analysis

Particle size distribution and concentration of OMVs were determined by nanoparticle tracking analyses (NanoSight NS300, Malvern Instruments, United Kingdom). Purified OMV samples were diluted up to a concentration acceptable for analysis within the recommended range of 100–120 visible particles per frame. Triplicate videos of each sample were taken using the equipped 532-nm green laser, and a high-sensitivity video with camera level of 14 was captured and performed using NanoSight 3.0 or 3.1 software, with a threshold of between 3 and 5. Triplicate video statistics were averaged for each sample. Further, the number of OMVs per milliliter was normalized to colony-forming unit (CFU) per milliliter of each strain to determine the number of OMVs per CFU (White et al., 2018). Approaches for determining size and production met the MISEV 2018 recommendations (Théry et al., 2018).

### Animals

Six- to eight-week-old female BALB/c mice were purchased from Dashou Biotechnology Co., Ltd., and were allowed to adapt to their new environment for 1 week before the animal experiments were initiated. Lohmann chickens (1 days old) were purchased from Muxing Poultry Co., Ltd. (Chengdu, China) and were maintained under standard conditions until they were 10 days old. All experiments involving animals were approved by Animal Use Ethical Committee of Nanchang University and were conducted in compliance with the Animal Welfare Act and related regulations (Hansen et al., 2017). The principles stated in the Guide for the Care and Use of Laboratory Animals were followed (National Research Council, 2011).

### Immunization Protocol and Challenge

Mice of approximately equal weights were divided into groups (*n* = 5 each) for intranasal (20 μg OMVs based on protein content in 10 μl DPBS per animal) and intraperitoneal immunizations (5 μg OMVs based on protein content in 100 μl DPBS per animal), which were performed two times without any adjuvant use. The negative control group was served the equivalent volume of DPBS. At 4 weeks, the doses were boosted. To determine the systemic and mucosal immunity induced by OMVs, blood samples of mice were collected *via* orbital sinus puncture, as well as vaginal secretions by repeatedly flushing the animals with 0.1 ml of PBS buffer at 2-week intervals after the first immunization. The blood serum and the supernatant of a vaginal wash were centrifuged and stored at −80°C for future use. To calculate the rate of protection, 5 weeks after the booster immunization, the mice were challenged with a lethal dose of *S.* Typhimurium S100 (1.1 × 10^9^ CFU, determined by continuously diluting and then counting on the LB plate) in 20 μl of buffered saline with 0.01% gelatin (BSG) *via* oral gavage. In another experiment, mice were immunized as above and then challenged with 1.3 × 10^7^ CFU of *S.* Choleraesuis (∼100-fold LD_50_), 1.2 × 10^7^ CFU of *S.* Enteritidis (∼100-fold LD_50_), 1.4 × 10^8^ CFU of APEC O78 (∼100-fold LD_50_), and 1.1 × 10^7^ CFU of *S. flexneri* 2a (∼100-fold LD_50_) in 20 μl of BSG 5 weeks after the booster immunization by oral gavage. Mice were monitored daily for weight, illness, and signs of obvious discomfort, distress, or pain. Mice that exhibited a weight loss of greater than 20% of their starting weight were euthanized *via* carbon dioxide followed by cervical dislocation. There were no expected deaths in this mice experiment.

To evaluate the protective efficacy of OMVs in chickens, 10-day-old Lohmann chickens were immunized intranasally (75 μg OMPs or OMVs in 100 μl DPBS per bird) at a 2-week interval as potential immune-protective administration, and 100 μl DPBS was used as a negative control. There were four groups of 20 chickens each (Wang et al., 2019). We marked the day of the first immunization as day 0 and the booster day as week 2. At weeks 1 and 4, 1–2 ml of blood was aseptically drawn from the chicken brachial vein after the first and second immunization. Then, we incubated the collected blood at room temperature for 30 min, removed the blood clot with sterile toothpicks, and centrifuged at 1,000 × *g* for 15 min. The supernatants were gently removed, and the prepared serum samples were stored at −80°C.

**Figure 1:**
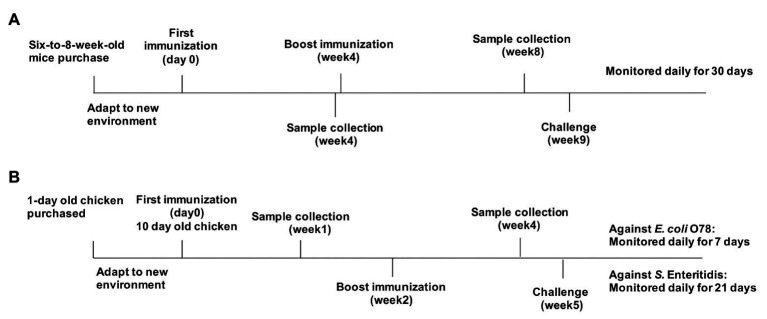
Immunization and challenge protocols for mouse and chicken experiments. **(A)** Mice were separately immunized with parental outer membrane vesicles (OMVs), OMVs from Δ*omp* mutants, or phosphate-buffered saline (PBS) on day 0, then boosted at week 4, and challenged at 5 weeks after boost immunization. Samples were collected at weeks 4 and 8. **(B)** Chickens were separately immunized with parental OMVs, K021 OMVs, bacterial OMVs, bacterial outer membrane proteins (OMPs), or PBS (control) on day 0, then boosted at week 2, and challenged at week 5. Blood samples were collected at weeks 1 and 4.

Protection of OMVs in chickens was evaluated in two different experiments. Experiment 1 was performed to determine the protection against wild type APEC O78 strain, and the immunized chickens were challenged *via* the air sac route with a lethal dose of the wild type APEC O78 strain (1.3 × 10^8^ CFU, ∼100-fold LD_50_ in chickens), which were suspended in 20 μl of BSG, at 3 weeks after booster administration (week 5). After challenge, the chickens were monitored for 7 days, and the clinical signs scored as follows: none = 0, reluctance to walk = 1, coughing, head shaking, and unwillingness to eat = 2, gasping, or lying down of a long time, or both, accompanied by ruffled feathers = 3, and death = 4 (Grgić et al., 2008). Dead chickens were necropsied immediately on the day of death. At the end of the 7 days of observation period, the surviving chickens were sacrificed and necropsied. Macroscopic lesions in the heart and liver were recorded and scored separately as follows: heart and pericardium (normal = 0, turbid with excessive or cloudy fluid in the pericardial cavity or partial pericarditis = 1, marked pericarditis = 2, and severe pericarditis or death = 3) and liver (normal = 0, small amount of fibrinous exudate = 1, marked perihepatitis = 2, and severe perihepatitis or death = 3; Nagano et al., 2012). Experiment 2 was performed to determine the protection against *S.* Enteritidis, and the immunized chickens were inoculated orally with 5 ml of DPBS containing 1 × 10^10^ CFU of *S.* Enteritidis at 3 weeks after booster immunization. LD_50_ of *S.* Enteritidis in chicken was not determined because this strain did not cause the death of chickens in our studies. Therefore, we monitored the fecal shedding of challenge strain to evaluate the protective efficacy against *S.* Enteritidis in chickens. Feces (5 g) were collected by rectal palpation and then were suspended in tetrathionate broth (TT; Oxiod, United Kingdom) and 10-fold serially-diluted suspensions were plated on brilliant green agar (Oxiod) supplemented with 1 μg/ml novobiocin and 50 μg/ml nalidixic acid (Oxiod). The colonies were counted after 24 h and five colonies were randomly selected for agglutination with anti-S. Enteritidis (ThermoFisher Scientific). The animal experiments were performed twice, and the data were combined for analysis (Figure 1).

### Enzyme-Linked Immunosorbent Assay

Outer membrane proteins were isolated from *Salmonella*, APEC, and *Shigella* as previously described (Carlone et al., 1986). IgG and IgA responses of mice were determined using a quantitative ELISA method, and the titers of anti-OMP IgG in chicken serum samples were determined by ELISA as described below. Briefly, NUNC MaxiSorp™ 96-well plates (Thermo Scientific, Waltham, MA, United States) were coated with 2 μg of OMPs from various serotypes of *Salmonella*, APEC, and *Shigella* per well in 100 μl of sodium carbonate/bicarbonate coating buffer (pH 9.6). The plates were then incubated at 4°C overnight. To construct standard curves for each antibody isotype and make the experimental data more accurate, plates were coated in triplicate with 2-fold dilutions of appropriate purified mouse Ig isotype standard IgG and IgA (BD Biosciences, San Jose, CA, United States), starting at 0.5 μg/μl. PBS containing 0.1% Tween 20 (PBST) was used to wash the plate and 2% bovine serum albumin was used to block the plate at room temperature. Subsequently, a 100-μl volume of a suitably diluted sample was added to wells and incubated for 1 h at room temperature. After washing the wells, biotinylated goat anti-mouse IgA and IgG (Southern Biotechnology, Inc., Birmingham, AL, United States) were added. The streptavidin-alkaline phosphatase conjugate (Southern Biotechnology, Inc.) was used to develop the protein signals and the *p*-nitrophenyl phosphate substrate (Sigma-Aldrich) was used to detect the signal of each well. Absorbance was read at 405 nm using an automated ELISA plate reader (iMark; Bio-Rad, United States). The final Ig isotype concentration of the serum samples for quantitative ELISA was calculated using appropriate standard curves, and a log-log regression curve was calculated from at least four dilutions of the isotype standards and used for ELISA.

### Opsonophagocytosis Uptake and Serum Bactericidal Activity Assay

The opsonophagocytosis assay was performed in serum samples to determine the function of OMV-raised antibodies, as previously described (Campbell and Edwards, 2000). RAW 264.7 macrophages (5 × 10^5^ cells/well) were seeded in 24-well plates and incubated in RPMI complete medium without antibiotics (500 μl/well; Gibco, United States) for 2 h at 37°C, 5% CO_2_ atmosphere. To perform opsonization with the antibody and complement system, a serum sample was diluted 1:100 and incubated with bacterial suspensions of 10^5^ CFU of APEC O78 and *S.* Enteritidis at 37°C for 10 min. To detect phagocytosis, we added the above opsonized bacteria to a macrophage monolayer in 24-well plates at a 1:10 (cells/bacteria) multiplicity of infection. The plates were incubated in an incubator at 37°C with 5% CO_2_ atmosphere for 30 min to allow phagocytosis of the bacterial cells by macrophages. One hundred microgram of gentamicin per ml cultured for 72 h was used to kill unattached extracellular bacteria, and the plates were washed three times with PBS. The intracellular bacteria were released by immediately lysing the macrophages with 1% Triton X-100 at 37°C for 10 min. The lysates were consecutively diluted 10-fold and plated onto LB plates, which were incubated at 37°C overnight. The numbers of phagocytosed bacteria were calculated by enumerating the colonies on the plates.

A modified serum bactericidal activity (SBA) assay was performed to evaluate the bactericidal ability of antibodies in serum samples *in vitro*. The serum was diluted 10-fold with LB medium. We cultured the wild type APEC O78 strain and *S.* Enteritidis to the logarithmic phase (OD_600_ = 0.7) and then suspended the cells in PBS at a concentration of 10^6^ CFU/ml. Ninety microliter of the diluted sera was mixed with 10 μl of the bacterial suspensions in the wells of 96-well plates and then incubated at 37°C with shaking at 100 rpm for 1 h. The mixture was consecutively diluted 10-fold and inoculated on plates at 37°C overnight, followed by counting the viable colonies. The survival rates of bacteria in different serum samples were calculated by dividing the CFU counts after serum treatment by the original CFU counts. To make the results more accurate, we independently tested all serum samples three times.

### Statistical Analysis

All statistical analyses were performed using the GraphPad Prism 5 software (GraphPad Software, San Diego, CA, United States). One- or two-way analysis of variance was performed followed by Tukey’s *post hoc* test to determine the significance of differences between the mean values of the experimental and control groups. Values are presented as mean ± standard deviation (SD). Differences in the survival rates among all groups were analyzed using the log-rank sum test. *p* < 0.05 was indicated statistically significant.

## Results

### Characterization and Quantification of OMVs

**Figure 2:**
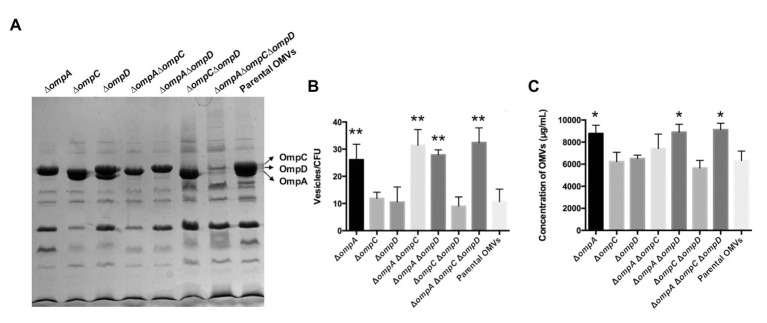
Characterization and quantities of OMVs derived from the ∆*omp* mutants and parental strain of *Salmonella* Typhimurium. **(A)** Protein contents in OMVs from the mutants and parental strain, analyzed by SDS-PAGE. The outer membrane proteins OmpC, OmpD, and OmpA are marked on the right. Strains (from left to right): K015 (∆*ompA*), K016 (∆*ompC*), K017 (∆*ompD*), K018 (∆*ompA*∆*ompC*), K019 (∆*ompA*∆*ompD*), K020 (∆*ompC*∆*ompD*), K021 (∆*ompA*∆o*mpC*∆*ompD*), and χ3761 (parent). **(B)** Numbers of particles per milliliter were normalized to numbers of CFU per milliliter calculated from each mutant and parental strain. **(C)** Concentrations of OMVs derived from the ∆*omp* mutants and parental strain (same order as in **A**). Data are presented as means ± standard deviations (SD) for three independent cultures. ^*^p < 0.05 and ^**^p < 0.01.

Outer membrane vesicles purified from strains K084, K015, K016, K017, K018, K019, K020, and K021 (all mutations were identified by PCR and gene sequencing, Supplementary Figure 1) were evaluated using protein profile assays. The major OMPs, including OmpA, OmpC, and OmpD, are marked in Figure 2A on the right, which were consistent with previous study (Liu et al., 2016a). Except the corresponding bands of the OmpC, OmpD, and OmpA proteins, the bands of other proteins in the protein profiles were basically the same. The results were consistent with the expected deficiency of certain proteins. Further, cryo-EM data showed that OMVs purified from the Δ*ompA*Δ*ompC*Δ*ompD* mutant strains were spherical, and no other contaminant was visible, which indicated that deletion of major OMPs did not affect the structure of OMV particles (Supplementary Figure 2). We also determined the particle size of OMVs and enriched fractions during density gradient purification process using nanoparticle tracking analysis (Théry et al., 2018). The results showed that the majority of the produced OMVs derived from *omp* mutant strains and parental strain had a diameter of 70–90 nm, and the highest concentration of particles was in consecutive fractions 25 and 30% OptiPrep (Supplementary Figures 3, 4), which indicated that the *omp* knockout mutants did not affect the particle size of OMVs secreted by the bacteria. Further, we observed a statistically significant increase in OMVs release from Δ*ompA*, Δ*ompA*Δ*ompC*, Δ*ompA*Δ*ompD*, and Δ*ompA*Δ*ompC*Δ*ompD* compared to that in the parental strain (Figure 2B). The results based on protein quantification of OMVs also confirmed this observation (Figure 2C). Moreover, the results of OMVs cultured in acidic minimal medium (MgM, pH 5.0) showed similar trends as those in LB medium (Supplementary Figure 5). Compared with that of the parental strain, the *omp* knockout mutants showed certain effects on the OMV yields both in LB and acidic MgM growth conditions, among which the *ompA* mutant strain had a significantly higher yield than did the parental strain, suggesting that Δ*ompA* might be beneficial for OMV production.

### IgG and IgA Immune Responses Elicited by OMVs Derived From the *∆omp* Mutants and Parental Strain Against *S.* Typhimurium

**Figure 3:**
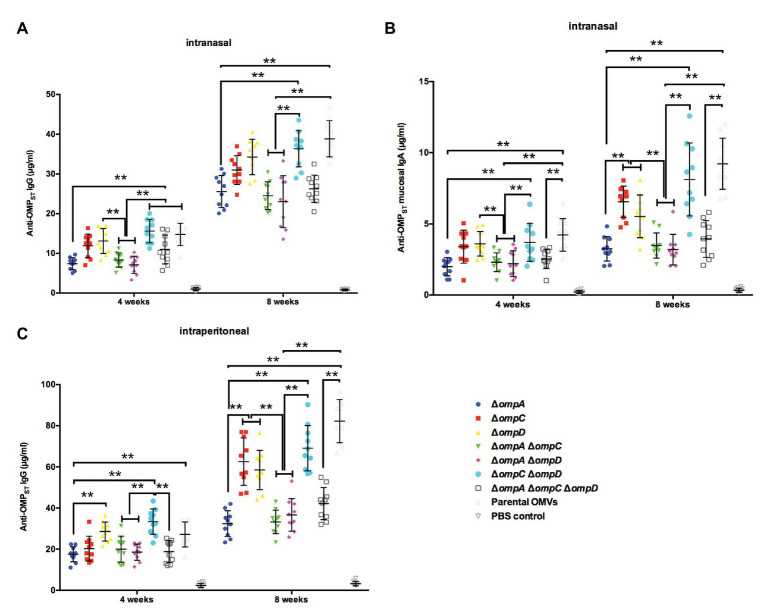
IgG and IgA immune responses in mice immunized with OMVs derived from the parental strain and Δ*omp* mutants. Total amounts of anti-OMP_ST_ IgG in the sera of mice immunized with OMVs *via* the intranasal **(A)** or intraperitoneal **(C)** route and total amounts of anti-OMP_ST_ IgA in vaginal secretions from mice immunized with OMVs *via* the intranasal route **(B)**. Samples were collected at 4 and 8 weeks after the first immunization, and PBS-treated mice served as the control group (*n* = 10 mice per group). The error bars represent variations among all mice in each group. ^**^p < 0.01.

To verify the immunogenicity of OMP-deficient OMVs, mice were intranasally and intraperitoneally injected with OMVs purified from the Δ*omp* mutants and the parental strain, and systemic and mucosal antibody levels were measured in the mice. When the mice were intraperitoneally injected, a small bump appeared in their abdominal cavity but disappeared in less than 12 h. All OMVs elicited lower levels of IgG response *via* the intranasal route than that *via* the intraperitoneal route at 4 weeks post-immunization, whereas those from the Δ*ompC*Δ*ompD* mutant caused a relatively higher production of serum IgG than did the other groups, except the parental OMVs, regardless of the administration route. After booster immunization, a relatively higher IgG level was observed in the group immunized with parental OMVs compared with the other groups; by contrast, the production of IgG from the Δ*ompC*Δ*ompD* OMV group was higher than those from the other groups of Δ*omp* mutants (Figures 3A,C). We also determined the levels of anti-OMP IgA in vaginal secretions from the mice as an indicator of mucosal response. After boosting, we could observe a sharp increase in the levels of mucosal IgA, with mice injected with OMVs from the Δ*ompC*Δ*ompD* mutant having higher levels than the mice immunized with the other OMVs from Δ*omp* mutants, which was consistent with the effect of IgG level (Figure 3B). Measurements of the IgA responses induced *via* the intraperitoneal route showed that the values of all groups were lower than 0.5 μg/ml, and no significant difference was observed. At 5 weeks after boost immunization, we infected the mice with 1 × 10^9^ CFU (approximately 2,000 × LD_50_) of wild type *S.* Typhimurium strain S100 to monitor the protective efficiency and recorded survival rates. Immunization with OMVs from the parental strain, Δ*ompC*Δ*ompD*, and Δ*ompA*Δ*ompC*Δ*ompD* mutants induced effective protection against the challenge. All the mice in the control (PBS) group succumbed within 10 days after infection with wild type *S.* Typhimurium strain S100 (Table 3).

**Table 3:** Immunization with distinct OMVs with OMP mutations protected mice against oral challenge with *S.* Typhimurium strain S100.

Groups	Number of surviving mice/total number of mice^*^	
Immunization administration	Intranasal route	Intraperitoneal route
K084 (parental strain)	4/5 (80%)	5/5 (100%)
K015 (∆*ompA*)	1/5 (20%)	1/5 (20%)
K016 (∆*ompC*)	3/5 (60%)	3/5 (60%)
K017 (∆*ompD*)	3/5 (60%)	4/5 (80%)
K018 (∆*ompA*∆*ompC*)	3/5 (60%)	4/5 (80%)
K019 (∆*ompA*∆*ompD*)	1/5 (20%)	3/5 (60%)
K020 (∆*ompC*∆*ompD*)	4/5 (80%)	5/5 (100%)
K021 (∆*ompA*∆*ompC*∆*ompD*)	4/5 (80%)	5/5 (100%)
PBS group	0/5 (0%)	0/5 (0%)

* All vaccine groups except groups of intranasal immunization of K015 (∆*ompA*) and K019 (∆*ompA* ∆*ompD*) were significantly different from the PBS-vaccinated group (*p* < 0.01). There was no significant difference in protection among the other groups.

### Cross-Reactive Immune Responses Against *Salmonella* of Heterologous Serotypes and Other Major Bacterial Pathogens

**Figure 4:**
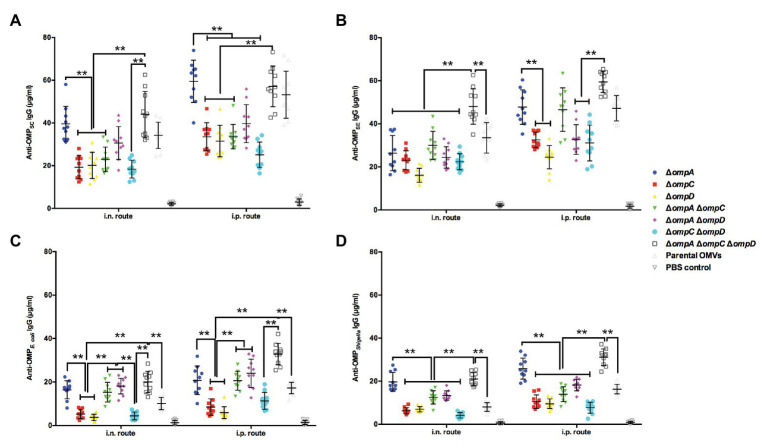
Cross-reactive immune responses induced by OMVs from Δ*omp* mutants against heterologous *Salmonella* and other intestinal pathogens. Total amounts of anti-OMP_SC_ IgG **(A)** anti-OMP_SE_ IgG **(B)** anti-OMP_O78_ IgG **(C)** and anti-OMP_Shigella_ IgG **(D)** in the sera of mice (*n* = 10 per group) immunized with OMVs *via* the intranasal or intraperitoneal route. The abscissa is the route of immunization, and the ordinate is the concentration of specific IgG. The error bars represent variations among all mice in each group. ^**^ *p* < 0.01.

Our previous studies have reported that truncation of the bacterial outer membrane may continuously expose conserved antigenic proteins to improve the cross-immune effect of OMVs (Liu et al., 2016a,b). Therefore, we evaluated the cross-immune responses of OMVs derived from different OMP mutants. In short, we determined the concentrations of IgG in the mice immunized with OMP-deficient OMVs and in those immunized with OMPs isolated from *S.* Enteritidis, *S.* Choleraesuis, APEC O78, and *Shigella* by a quantitative ELISA to evaluate the effects of cross-immunity. The result for the two routes of immunization showed that the IgG concentrations were roughly the same, as both routes resulted in high IgG levels against OMPs derived from *S.* Choleraesuis, *S.* Enteritidis, APEC O78, and *Shigella*. Regardless of the immunization route, analysis and integration of the data showed that the anti-OMP_SC_ IgG concentrations in the Δ*ompA*Δ*ompC*Δ*ompD* and Δ*ompA* groups were slightly higher or similar to those in the parental OMV group *via* intranasal or intraperitoneal immunization, respectively (Figure 4A). Regarding *S.* Enteritidis, the result of cross-immunity in the Δ*ompA*Δ*ompC* group showed that the level was close to that in the group of parental OMVs; only the Δ*ompA*Δ*ompC*Δ*ompD* group performed better than did the parental OMVs group (Figure 4B). OMVs from the Δ*ompA*, Δ*ompA*Δ*ompC*, Δ*ompA*Δ*ompD*, and Δ*ompA*Δ*ompC*Δ*ompD* mutants elicited remarkedly higher levels of serum IgG than did the parental OMVs against OMPs from APEC O78 (Figure 4C). A significant increase in IgG levels against *Shigella* OMPs was observed in the sera from the mice immunized with OMVs from the Δ*ompA* and Δ*ompA*Δ*ompC*Δ*ompD* mutants compared with those in the sera from the mice immunized with OMVs from the parental strain (Figure 4D). Both intranasal and intraperitoneal injections resulted in a concentration of IgG that was half that of the homologous group but still showed a good protective efficacy.

### Cross-Protection Against *Salmonella* of Heterologous Serotypes and Other Major Bacterial Pathogen Challenges

**Figure 5:**
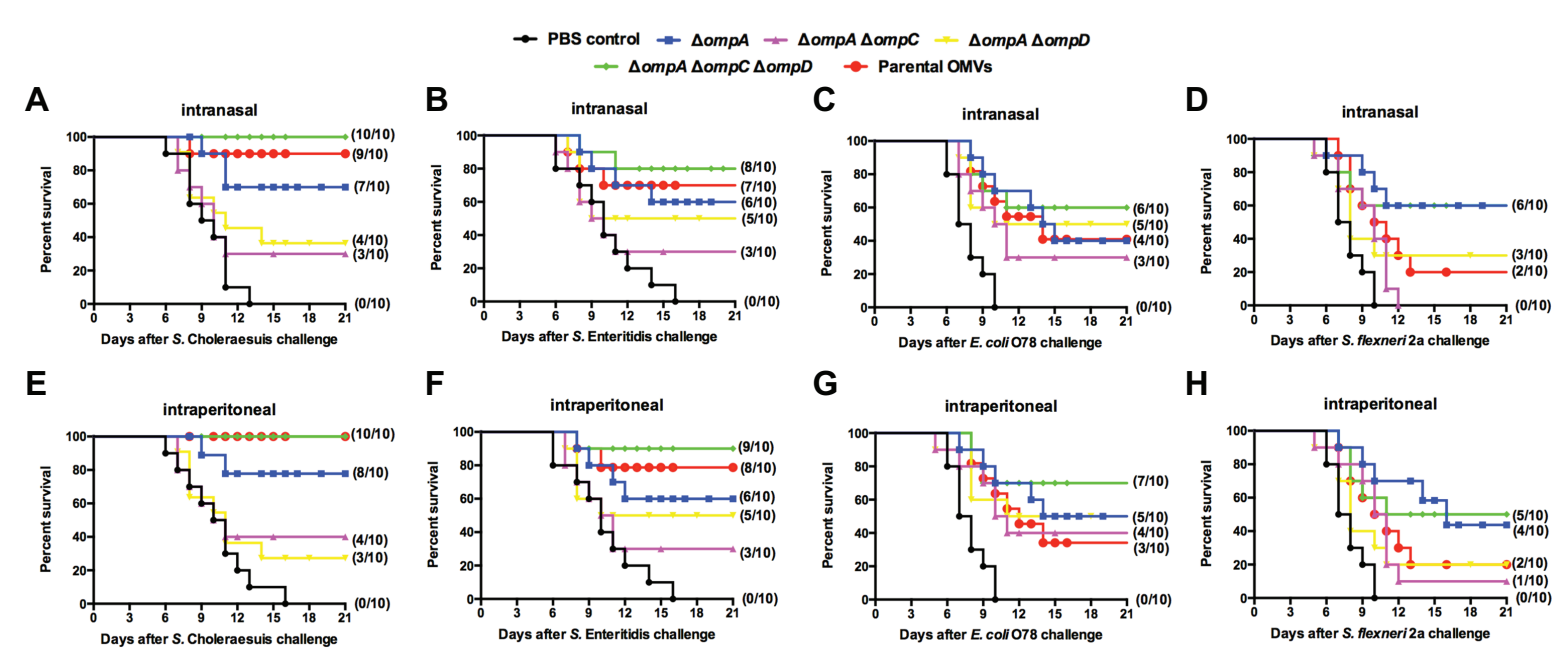
Cross-protection induced by OMVs from Δ*omp* mutants against challenge with heterologous-serotype *Salmonella* and other intestinal pathogens. Mice were immunized intranasally **(A–D)** or intraperitoneally **(E–H)** with OMVs isolated from the indicated *Salmonella* mutants. The immunized mice were orally challenged with *S.* Choleraesuis **(A,E)** *S.* Enteritidis **(B,F)** APEC O78 **(C, G)** and *Shigella flexneri* 2a **(D,H)** 9 weeks after the first immunization. After the challenge, mortality was monitored every 3 weeks. The number of surviving mice/total number of mice (*n* = 10 per group) is shown in parentheses.

To assess cross-protective productivity, mice from different groups were immunized with OMVs from the Δ*ompA*, Δ*ompA*Δ*ompC*, Δ*ompA*Δ*ompD*, and Δ*ompA*Δ*ompC*Δ*ompD* mutants *via* the intraperitoneal and intranasal routes, with the PBS group used as a control. Similar to the experiments on cross-reactivity, mutant strains with a better efficacy were selected to explore their potential as cross-protective vaccines. In the case of *Salmonella* with different serotypes, the groups immunized with OMVs derived from various OMP mutants all showed good cross-protection, regardless of the route of immunization, compared with that in the PBS group (Figure 5A,B). In particular, the group immunized with Δ*ompA*Δ*ompC*Δ*ompD* OMVs *via* the intraperitoneal route showed better or similar protection than did the parental group (Figures 5E,F). Even upon challenge with APEC O78 (Figures 5C,G) and *Shigella* (Figures 5D,H), Δ*ompA*Δ*ompC*Δ*ompD* OMVs still provided an established protection efficiency compared with OMVs from the parental strain and other Δ*omps* mutant strain. Irrespective of the wild type strain used as a challenge and the immunization route, OMVs from the Δ*ompA*Δ*ompC*Δ*ompD* mutant strain remained highly protective, and the survival rate of mice remained above 50%. In the case of the homologous bacterial infection, the survival rate even exceeded 80%.

### Cross-Immunogenicity and Protection Effects of *omp*-Deficient OMVs Against *S.* Enteritidis Challenge in the Chicken Model

**Figure 6:**
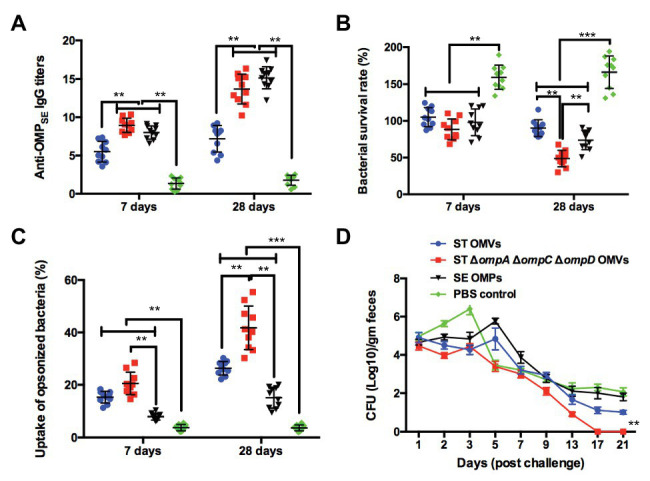
Cross-immunogenic protection with Δ*omp* OMVs against *S.* Enteritidis challenge in the chicken model. **(A)** Titers of anti-OMP IgG in the sera of chickens immunized with OMVs, OMPs, or PBS. **(B)** Bacterial survival after incubation at 37°C for 1 h with serum samples from PBS-, *S.* Enteritidis OMP-, *S.* Typhimurium OMV-, and K021 OMV-immunized chickens. **(C)** The phagocytosis rate of the phagocytic bacteria was obtained by comparing the phagocytosis of macrophage RAW 264.7 on the inoculated macrophages. **(D)** Chickens were challenged with 10^10^ CFU of *S.* Enteritidis at 3 weeks after the boost immunization. Fecal shedding of the challenge strain in animal feces, and each point on the graph represents mean fecal shedding along with the corresponding standard deviation for each group of animal for the day of fecal sampling. The error bars represent variations among all mice in each group. ^**^p < 0.01 and ^***^p < 0.001.

Based on the results of the previous experiments in the mouse model and the current status of *S.* Enteritidis as a major food-borne pathogen, we further studied the immunity of mutant vaccine to *S.* Enteritidis in a chicken model. To verify the cross-immunogenicity of OMVs from the Δ*ompA*Δ*ompC*Δ*ompD* mutant, which could induce high levels of cross-protection and high survival rates after the challenge in mice, chickens were intranasally immunized with purified OMVs from strain K021 and the parental strain (K084), as well as with OMPs isolated from *S.* Enteritidis, as potential immune-protective injections. At weeks 1 and 4, the anti-OMP_SE_ IgG was at a high level in the Δ*ompA*Δ*ompC*Δ*ompD* OMVs group, which was consistent with that of SE OMPs group (Figure 6A). In the period between week 1 and week 4, the bacterial survival rates declined, and at week 4, levels of Δ*ompA*Δ*ompC*Δ*ompD* OMVs group were significantly decreased compared with the SE OMP group, which indicated that booster immunization with OMVs from the Δ*ompA*Δ*ompC*Δ*ompD* mutant could significantly enhance SBA and display a long-term bacterial killing or growth suppression effect (Figure 6B). Compared with that of the PBS- and SE OMP-immunized groups, a significantly higher capability for opsonophagocytosis was shown at all time points (weeks 1 and 4) by the parental OMV- and Δ*ompA*Δ*ompC*Δ*ompD* OMV-immunized groups (Figure 6C). Immunized chickens in the different groups were challenged *via* the air sac route with 1 × 10^10^ CFU of SE at week 5 to evaluate the cross-protective efficiency. As shown in Figure 6D, a significant level of reduction in shedding was observed in the Δ*ompA*Δ*ompC*Δ*ompD* OMVs-immunized group of chickens, and birds were negative by day 17 post-challenge. Moreover, the Δ*ompA*Δ*ompC*Δ*ompD* OMVs-immunized group could shed higher levels of the challenge strain compared with that the parental OMVs immunized group and SE OMP-immunized group (Figure 6D).

### Cross-Immunogenicity and Protection of *omp*-Deficient OMVs Against APEC O78 Challenge in the Chicken Model

**Figure 7:**
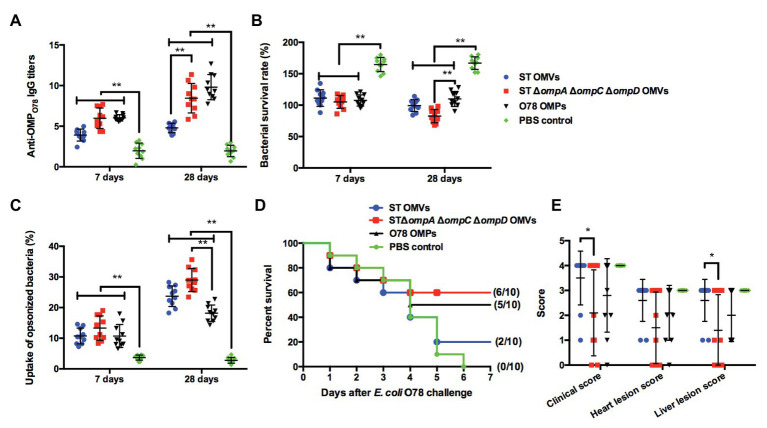
Cross-immunogenic protection with Δ*omp* OMVs against APEC O78 challenge in the chicken model. **(A)** Titers of anti-OMP_SE_ IgG in the sera of chickens intramuscularly immunized with purified OMVs from strain K021, the parental strain (K084), as well as with O87 OMPs and PBS. **(B)** Bacterial survival after incubation at 37°C for 1 h with serum samples from PBS-, O87 OMP-, *S.* Typhimurium OMV-, and K021 OMV-immunized chickens. **(C)** The phagocytosis rate of the phagocytic bacteria was obtained by comparing the phagocytosis of macrophage RAW264.7 on the inoculated macrophages. **(D)** Chickens were challenged with 10^8^ CFU of APEC O78 at 3 weeks after the boost immunization, and mortality was monitored for 7 days after the challenge. **(E)** Clinical features and tissues were scored for signs of colibacillosis. The error bars represent variations among all mice in each group. ^*^p < 0.05 and ^**^p < 0.01.

Further, we also determined the cross-immunogenicity of Δ*ompA*Δ*ompC*Δ*ompD* OMVs to APEC O78 in chickens and evaluated their cross-protective efficacy. At the first week, the production of anti-OMP_O78_ IgG in all experimental groups exhibited levels significantly higher than that in the PBS group. At week 4, the titers of anti-OMP_O78_ IgG in the Δ*ompA*Δ*ompC*Δ*ompD* OMVs group showed a similar trend as in the O78 OMP group, and were significantly higher than those in the parental group (Figure 7A). In addition, SBA assay showed that the bacterial survival rate of all experimental groups was significantly lower than that of PBS group at the first week; all experimental groups maintained this trend at the 4th week, although the Δ*ompA*Δ*ompC*Δ*ompD* OMVs group showed significantly lower bacterial survival rate than O78 OMP group (Figure 7B). Further, the bacterial uptake rate in the Δ*ompA*Δ*ompC*Δ*ompD* OMVs-immunized group was significantly higher than that in the O78 OMP group (Figure 7C). The post-challenge survival rate in the Δ*ompA*Δ*ompC*Δ*ompD* OMVs group approached 60%, higher than other groups, even in the O78 OMP group (Figure 7D). Moreover, immunization with Δ*ompA*Δ*ompC*Δ*ompD* OMVs provided significant reductions in clinical score compared to the parental and PBS groups. Similarly, liver lesion score was lesser in the Δ*ompA*Δ*ompC*Δ*ompD* OMVs immunized chickens than in the control and PBS groups (Figure 7E). Although there was no significant difference in the heart lesion score among the experimental groups, slightly lower score was observed in the Δ*ompA*Δ*ompC*Δ*ompD* OMV-immunized group compared with parental and O78 OMP groups. The relatively decreased clinical scores and liver lesion scores demonstrated that immunization of Δ*ompA*Δ*ompC*Δ*ompD* OMVs could lower the severity of perihepatitis induced by APEC O78 in the birds (Nagano et al., 2012). Taken together, these data clearly demonstrated the feasibility of using Δ*ompA*Δ*ompC*Δ*ompD* OMVs as a broad-spectrum vaccine.

## Discussion

Outer membrane vesicle-based vaccines show several potential advantages over live attenuated vaccines, including a high immunogenicity without replication, a higher safety, and better intrinsic adjuvant effects, thus being a novel choice to be used against bacterial infections (Roy et al., 2010, 2011; McConnell et al., 2011; Mitra et al., 2012). For example, OMVs from *Neisseria meningitidis*, which contains three highly immunogenic proteins, have been used as a licensed vaccine in children older than 2 months in Europe (Villena et al., 2018). However, until now, there is no licensed OMV vaccine for the protection against avian diseases. OMVs are composed of OMPs, LPS, lipoproteins, and some other components (Kuehn and Kesty, 2005). Our previous studies have demonstrated that OMVs derived from a Δ*fliC*Δ*fljB* mutant could induce the production of cross-reactive antibodies but did not affect the production of OMVs (Liu et al., 2016a), whereas deletion of the *rfbP* gene, which induces a lack of full length O-antigen in LPS, could result in remodeling of the outer membrane structure and, thus, enhance the production of OMVs (Liu et al., 2016b). Based on these previous findings, we continued studying the effects of knockout of several major OMPs (OmpA, OmpC, and OmpD) on the cross-protection with OMVs and testing our hypothesis that remodeling of the outer membrane might affect the cross-protection with OMVs. Our results showed that Δ*ompA* could increase the yield of OMVs, and Δ*ompA*Δ*ompC*Δ*ompD* OMVs provided good cross-protection against heterologous *Salmonella* and APEC in chickens, which was in line with our hypothesis.

The formation of OMVs is an important means of resistance of bacteria to diverse external environments, and various triggers, including iron limitation, antibiotics, signaling molecules, DNA-damaging agents, and hydrophobic compounds, can induce membrane vesicle formation through membrane blebbing or explosive cell lysis (McBroom et al., 2006; Toyofuku et al., 2019). The production of vesicles varies depending on the growth period and the availability of nutrients, and vesicle-related enzymes may contribute to the elimination of nutrients. The yield of OMVs is a critical factor that limits their application for clinical treatment. The knockout of OMPs may affect the biosynthesis of the outer membrane and thus the yield of OMVs (van de Waterbeemd et al., 2010; Nevermann et al., 2019). Our results also support this hypothesis, as Δ*ompA*, Δ*ompA*Δ*ompD*, and Δ*ompA*Δ*ompC*Δ*ompD* mutations could increase the OMV production (Figure 2B). On the contrary, the Δ*ompC*Δ*ompD* mutant produced fewer OMVs than did the parental strain. We speculated that the knockout of the *ompA* gene might affect the formation of the outer membrane, increasing the outer membrane vesiculation and content.

The development of an ideal vaccine to control bacterial infections should take systemic, mucosal, and cellular immunity into consideration (Pasetti et al., 2003; Capozzo et al., 2004). Therefore, both intranasal and intraperitoneal routes of administration to mice were chosen to evaluate the mucosal and systemic immunogenicity of OMVs isolated from OMP-deficient *S.* Typhimurium. Researchers have paid much attention to the intranasal route for vaccination (Partidos, 2000), which has previously been shown to be a good choice to elicit active systemic and mucosal immunity (Kiyono and Fukuyama, 2004; Holmgren and Czerkinsky, 2005). The intraperitoneal route, which can induce a strong humoral immunity, is also a standard route of immunization of mice with purified OMVs (Alaniz et al., 2007; Schild et al., 2009; Baker et al., 2017). In our previous study, both intranasal and intraperitoneal routes resulted in good immunogenic effects. Moreover, intranasal immunization may be a safer route for vaccination with OMVs (Liu et al., 2016a). Consequently, these two routes of immunization were used to assess the immunogenicity and protection ability of OMVs in this study. As shown in Figures 3, 5, although the ability of intranasal route to induce IgG production was not as strong as that of intraperitoneal route, intranasal route can effectively induce mucosal immune response, and intranasal OMV immunization could also elicit cross-protection similar to that of intraperitoneal immunization. This also confirmed that the intranasal route may be the preferred immunization strategy for OMVs as vaccines.

To assess the cross-protection provided by OMVs derived from OMP-deficient *S.* Typhimurium, it is necessary to ensure that the amount of various OMVs we immunized was consistent. With the deletion of the OMVs from the main OMPs, a question we considered was whether the cross-protection of Δ*ompA*Δ*ompC*Δ*ompD* OMVs achieved is a function of the estimated OMVs amount as indicated by protein concentration measurement. Therefore, we analyzed the particle size and distribution of each fractions of various OMP-deficient OMVs obtained by density gradient centrifugation using NTA, and found that the size and appearance of OMVs secreted by different OMP mutations were similar (Supplementary Figures 3, 4), which preliminarily indicated that the amount of OMVs can be accurately estimated by protein concentration measurement. And that, based on the cross-protection data of the chicken model, OMVs derived from OMP mutations stimulated mice to produce the same or even higher immune protection compared with the OMPs isolated from heterologous pathogen against this heterologous pathogen infection (Figures 6, 7). This also indicated that the cross-protection is not caused by the amount of immunized OMVs, but more likely by an increase in the expressed cross-immunogenic antigens. Taken together, these results confirmed that the number of OMVs from various OMP mutations that we immunized into mice is consistent, and the results of cross-protection induced by Δ*ompA*Δ*ompC*Δ*ompD* OMVs are also reliable.

In this study, *S.* Choleraesuis, *S.* Enteritidis, APEC O78, and *S. flexneri* 2a, which had been isolated from infected animals, were chosen to assess the immune efficiency of purified OMVs in mice. Owing to the deficiency of bacterial protective OMPs, the IgG levels against these pathogenic bacteria were higher in all groups than in the negative control group, especially in the Δ*ompA*Δ*ompC*Δ*ompD* OMVs group, wherein the levels were also obviously higher than those in the parental group (Figures 4A–D). Furthermore, in the mouse model, Δ*ompA*Δ*ompC*Δ*ompD* OMVs also provided appreciable protection against heterologous serotypes of *Salmonella*, including *S.* Choleraesuis and *S.* Enteritidis, APEC O78, and even human pathogenic *S. flexneri* 2a (Figure 5), which can cause significant diarrheal disease and mortality in humans (Groisman and Ochman, 1993). Recently, studies have reported that the O-antigen of *Shigella* was synthesized in *Salmonella* to construct a cross-protective vaccine to multiple disease-predominant *S. flexneri* serotypes, which suggested that *Shigella* and *Salmonella* had similar O-antigen synthesis pathways (Dharmasena et al., 2017; Su et al., 2019). Meanwhile, this fact might partly explain the cross-protection by these OMP-deficient OMVs against *Shigella* infection and also suggested that we could recombine the O-antigen of *Shigella* into OMVs to construct a cross-protective vaccine against predominant causative agents of shigellosis.

Avian pathogenic *Escherichia coli* O78 infections result in a great harm to young chickens, causing avian colibacillosis and even deaths, reducing the profit of the breeding industry. Although *S.* Enteritidis infection does not cause death in poultry, *S.* Enteritidis is a major cause of food-borne diseases, and poultry-derived products, including meat and chicken eggs, are considered a major source of this pathogen in human infection (Bäumler et al., 2000). Vaccine could be useful for preventing *S.* Enteritidis circulation in chickens and diminishes its transmission to humans. Therefore, we also evaluated the cross-protection of the OMP-deficient OMVs against *S.* Enteritidis and APEC O78 in a chicken model. The results showed that Δ*ompA*Δ*ompC*Δ*ompD* OMVs provided an immune effect, which was significantly higher than the immune effects of OMPs from *S.* Enteritidis and APEC O78. Thus, the data indicated that the Δ*ompA*Δ*ompC*Δ*ompD* OMVs could improve the cross-protection effectiveness against APEC O78 and feces shedding against *S.* Enteritidis (Figures 6, 7). Thus, OMVs derived from the Δ*ompA*Δ*ompC*Δ*ompD* mutations might also be effective and promising adjuvants in a vaccine design for chickens to prevent infections.

Currently, it is not clear whether the enhanced cross-protection induced by OMVs can be attributed to the conserved OMPs or to other conserved antigens that are shared among these pathogens, such as the LPS core oligosaccharide moiety that is possessed by various Gram-negative bacteria (Yang et al., 2013). Previous study reported that conserved LPS oligosaccharides in OMVs also contribute to inducing protective immunity (Lorenzo et al., 2015). However, these sugar epitopes may provide limited broad-spectrum protection against smooth Gram-negative bacterial infections (Francis and Shenep, 1985; Siber and Warren, 1985). Therefore, we can preliminarily speculate that the deletion of major OMPs might have increased the expression levels of conserved OMPs or other conserved antigens, which are typically found in the four enteropathogenic bacteria in this study, leading to the enhancement of cross-protection. Nevertheless, the existing data could not clarify which component causes the variation in immunogenicity. In the future, we will advance our research *via* direct identification, using proteomic analysis, of the conserved antigens that lead to the increase of cross-protection by OMVs. Moreover, OMVs can encapsulate antigenic components of other pathogens and serve as a delivery platform to induce desired pathogen-specific immune responses, thus expanding the application of OMVs as vaccines (Gnopo et al., 2017). Therefore, our future efforts would focus on OMVs as a multiple antigen platform to encapsulate conserved antigens or other O-antigens for the development of a broad-spectrum protective vaccine against avian pathogenic bacteria.

In conclusion, we discovered that the deletion of the *ompA* gene could enhance the production of OMVs and that OMVs derived from the Δ*ompA*Δ*ompC*Δ*ompD* mutations might be an effective and promising vaccine candidate for chickens to prevent *S.* Enteritidis and APEC O78 infections. OMP-deficient OMVs may not only be nominated as an effective vaccine candidate but may also present a novel vaccine adjuvant, which can be formulated with other vaccine components to elicit multi-serotype cross-protection responses and control infection with heterologous bacterial serotypes.

## Data Availability Statement

The raw data supporting the conclusions of this article will be made available by the authors, without undue reservation.

## Ethics Statement

The animal study was reviewed and approved by Animal Use Ethical Committee of Nanchang University.

## Author Contributions

QL and XH conceived and designed the experiments. YC, KJ, BL, HY, HR, and JW performed the experiments. YC and KJ analyzed the data. YC, KJ, XH, and QL wrote the manuscript. All authors contributed to the article and approved the submitted version.

### Conflict of Interest

The authors declare that the research was conducted in the absence of any commercial or financial relationships that could be construed as a potential conflict of interest.

## Supplementary Material

Supplementary Figure 1 Identification of outer membrane protein (OMP) mutations by PCR. Strains include K015 (∆*ompA*), K016 (∆*ompC*), K017 (∆*ompD*), K018 (∆*ompA*∆*ompC*), K019 (∆*ompA*∆*ompD*), K020 (∆*ompC*∆*ompD*), and K021 (∆*ompA*∆o*mpC*∆*ompD*).

Supplementary Figure 2 Cryo-EM imaging of outer membrane vesicles (OMVs). OMVs derived from the ∆*ompA*∆o*mpC*∆*ompD* mutant strains of *Salmonella* Typhimurium were visualized using cyro-EM. The red arrows indicate the visible OMVs.

Supplementary Figure 3 Distribution of OMV sizes from the ∆*omp* mutants and parental strain, binned in 10-nm increments. *n* = 3, mean ± SD.

Supplementary Figure 4 Graph representing particle numbers and mean particle size (Théry et al., 2018) for the OMVs derived from the ∆*omp* mutants and parental strain in various OptiPrep density gradient medium fractions, after NTA measurement. *n* = 3, mean ± SD.

Supplementary Figure 5 Numbers of particles per milliliter were normalized to numbers of CFU per milliliter calculated from each mutant and parental strain cultured in acidic MgM growth condition.

## Abbreviations

APEC: Avian pathogenic *Escherichia coli*
BSG: Buffered saline with 0.01% gelatin
CFU: Colony-forming units
DPBS: Dulbecco’s phosphate-buffered saline
LB: Luria-Bertani
LPS: Lipopolysaccharide
OMP: Outer membrane protein
OMV: Outer membrane vesicle
PBS: Phosphate-buffered saline
PBST: Phosphate-buffered saline containing 0.1% Tween 20
SBA: Serum bactericidal activity
SDS-PAGE: Sodium dodecyl sulfate polyacrylamide gel electrophoresis
