# Supplementary figures and images for: Immunization With Outer Membrane Vesicles Derived From Major Outer Membrane Protein-Deficient Salmonella Typhimurium Mutants for Cross Protection Against Salmonella Enteritidis and Avian Pathogenic Escherichia coli O78 Infection in Chickens
Source: Front Microbiol. 2020 Nov 3;11:588952. doi: 10.3389/fmicb.2020.588952 (PMC7720508; doi:10.3389/fmicb.2020.588952)

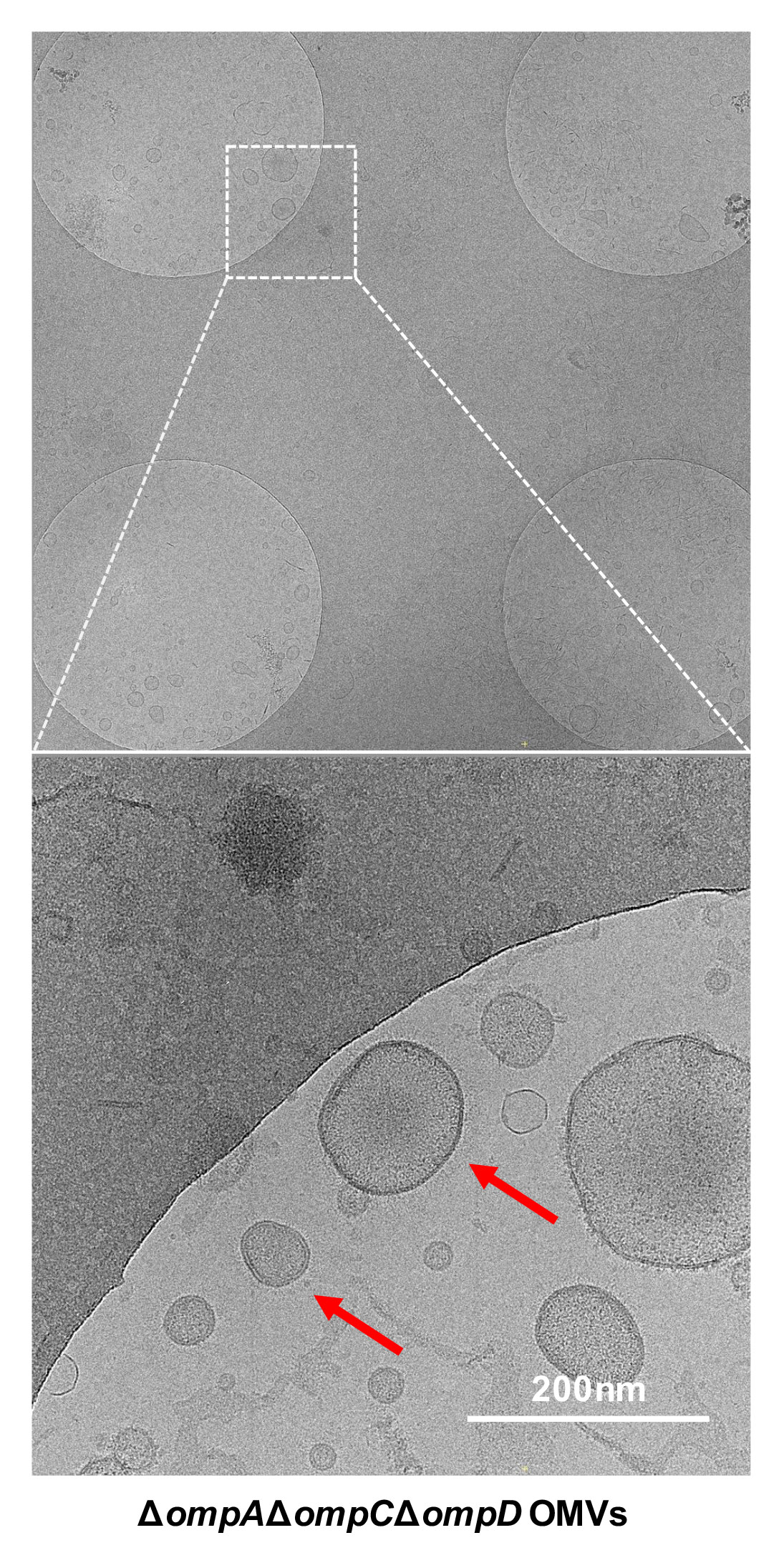

Supplement: Supplementary Figure 1 — Identification of outer membrane protein (OMP) mutations by PCR. Strains include K015 (∆ompA), K016 (∆ompC), K017 (∆ompD), K018 (∆ompA∆ompC), K019 (∆ompA∆ompD), K020 (∆ompC∆ompD), and K021 (∆ompA∆ompC∆ompD). [file Image_1.tif]

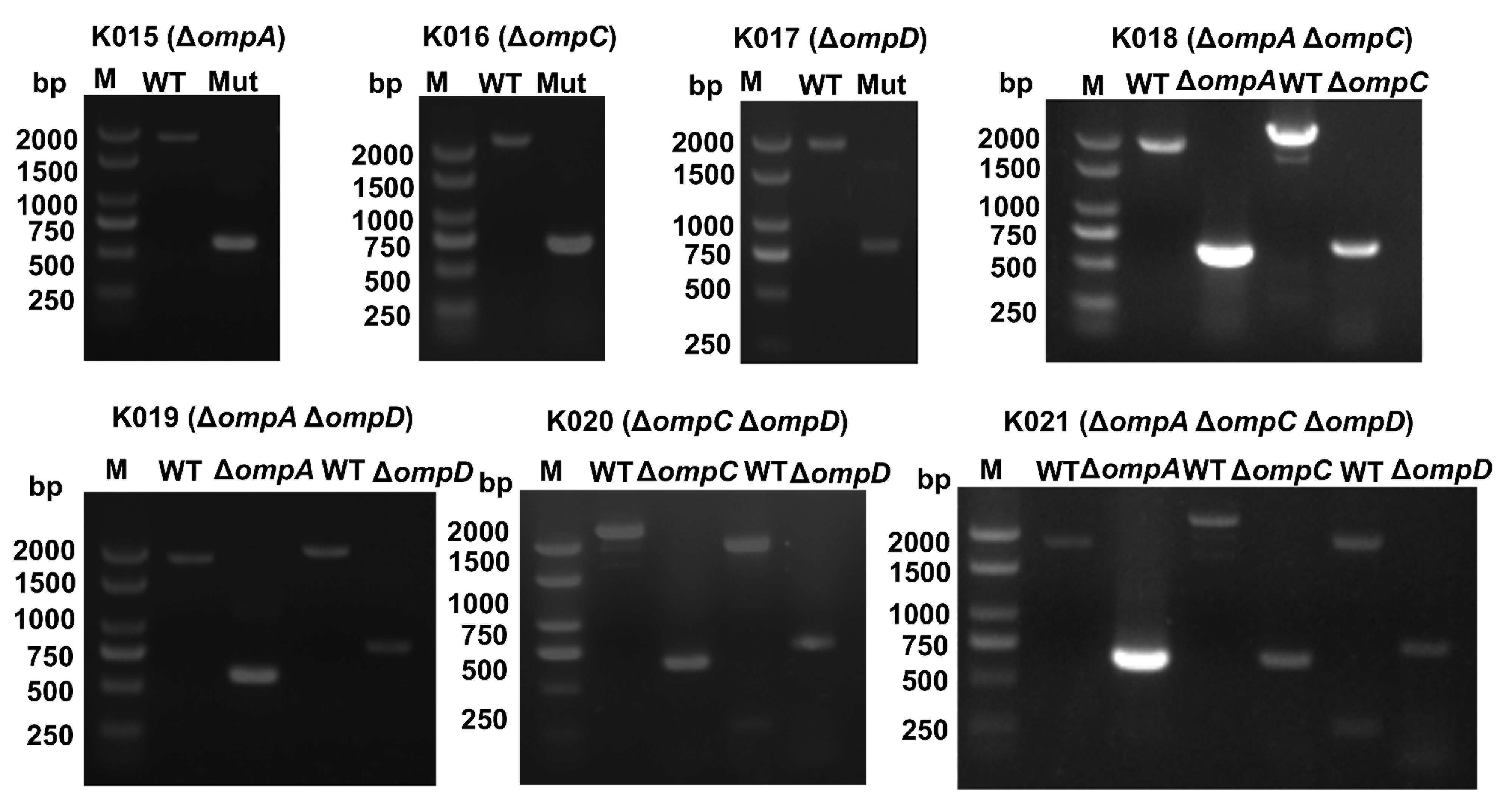

Supplement: Supplementary Figure 2 — Cryo-EM imaging of outer membrane vesicles (OMVs). OMVs derived from the ∆ompA∆ompC∆ompD mutant strains of Salmonella Typhimurium were visualized using cyro-EM. The red arrows indicate the visible OMVs. [file Image_2.tif]

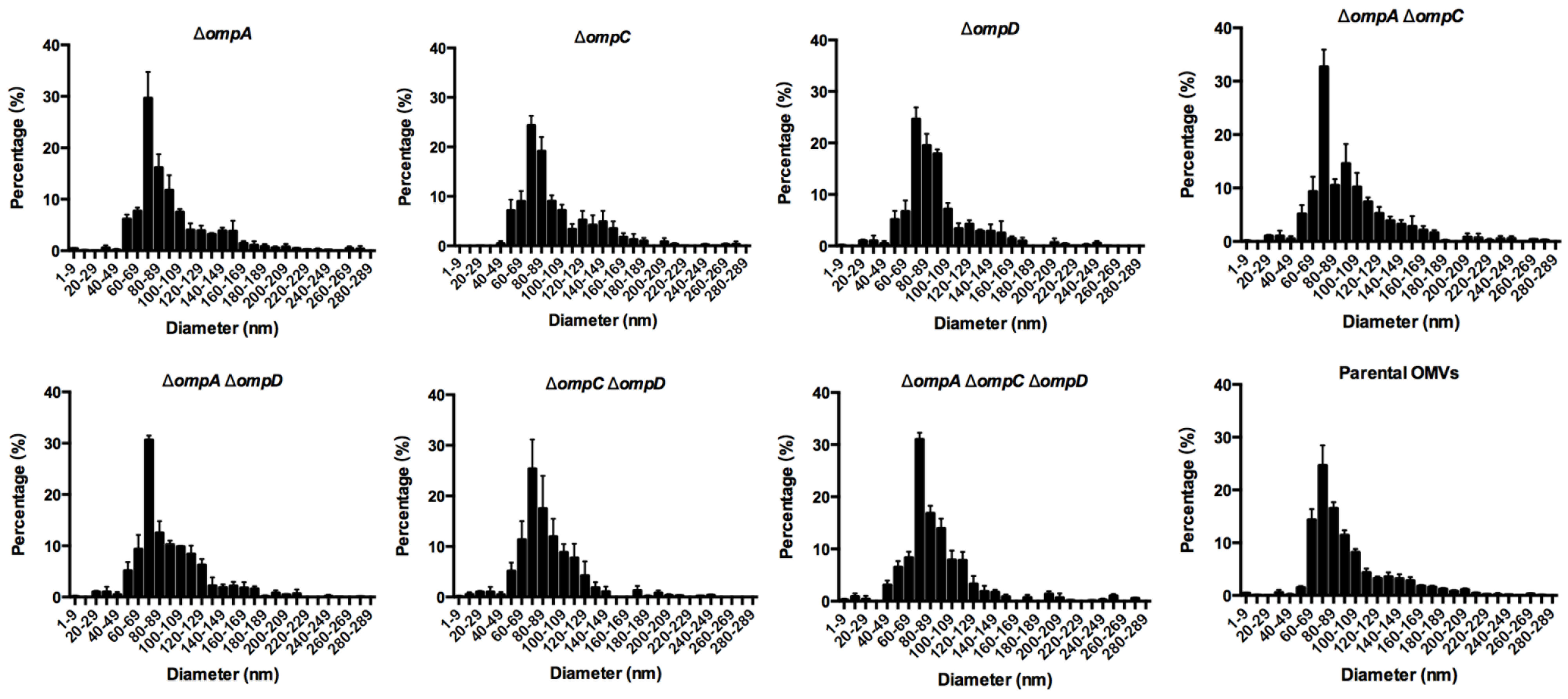

Supplement: Supplementary Figure 3 — Distribution of OMV sizes from the ∆omp mutants and parental strain, binned in 10-nm increments. n = 3, mean ± SD. [file Image_3.tif]

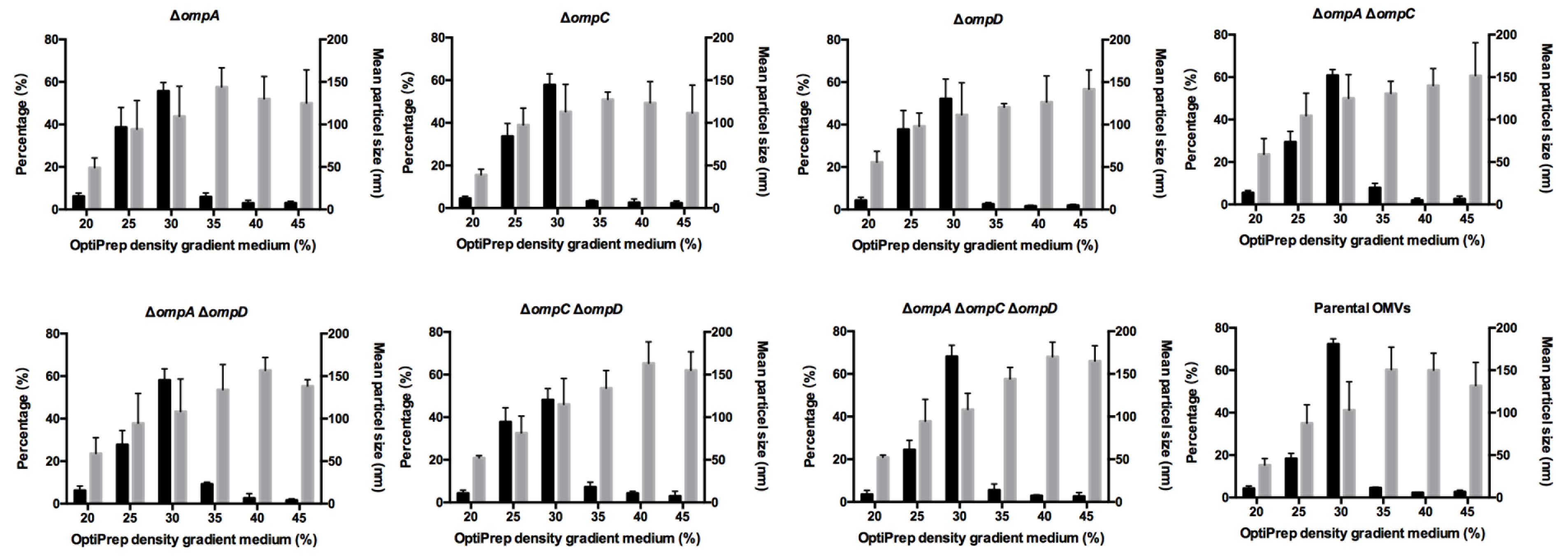

Supplement: Supplementary Figure 4 — Graph representing particle numbers and mean particle size (Théry et al., 2018) for the OMVs derived from the ∆omp mutants and parental strain in various OptiPrep density gradient medium fractions, after NTA measurement. n = 3, mean ± SD. [file Image_4.tif]

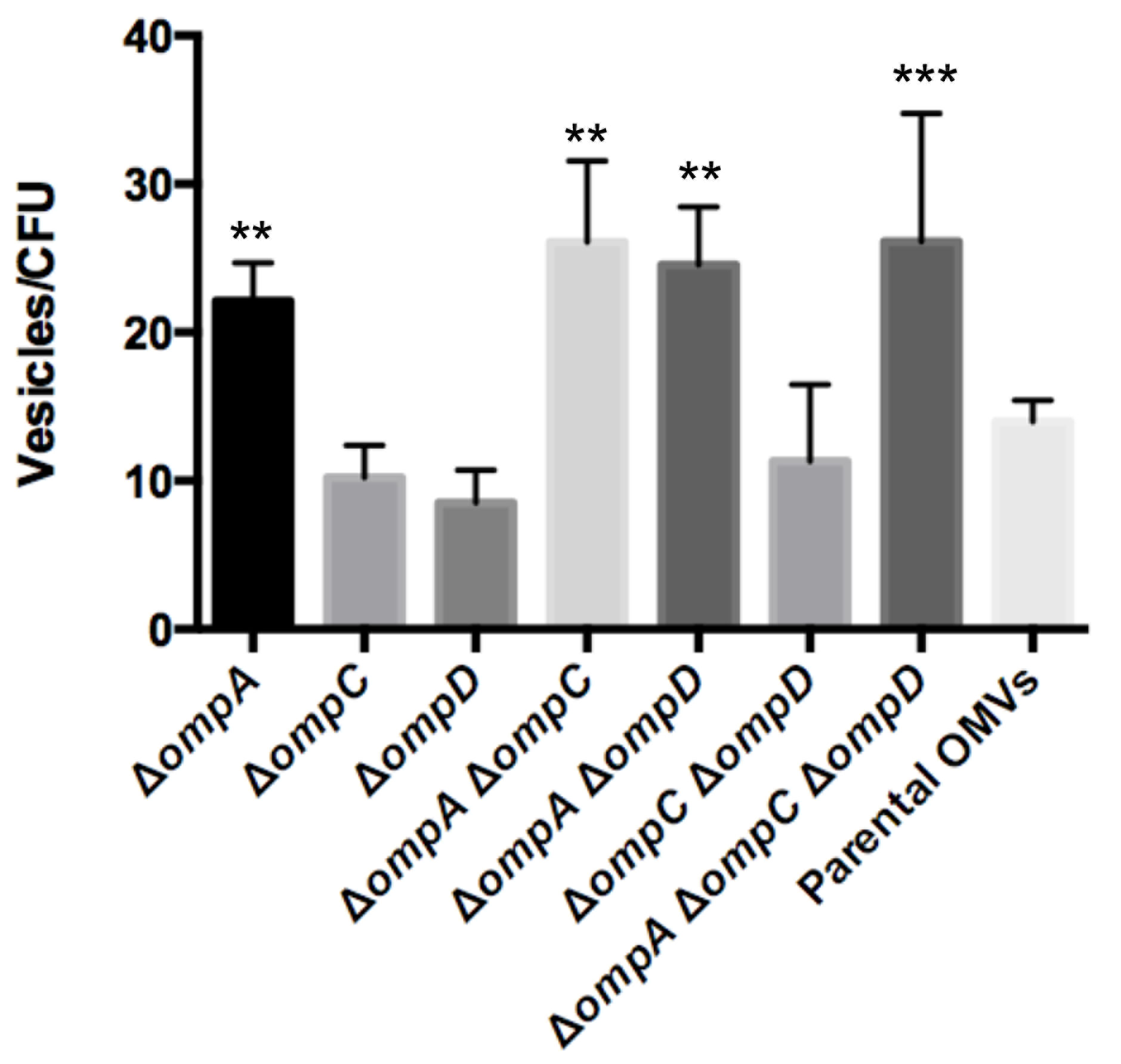

Supplement: Supplementary Figure 5 — Numbers of particles per milliliter were normalized to numbers of CFU per milliliter calculated from each mutant and parental strain cultured in acidic MgM growth condition. [file Image_5.tif]
